# Computational analysis and predictive modeling of polymorph descriptors

**DOI:** 10.1186/1752-153X-7-23

**Published:** 2013-02-04

**Authors:** Yugyung Lee, Sourav Jana, Gayathri Acharya, Chi H Lee

**Affiliations:** 1 School of Computing and Engineering, University of Missouri-Kansas City, Missouri, MO, 64110, USA; 2 Division of Pharmaceutical Sciences, College of Pharmacy, University of Missouri-Kansas City, Missouri, MO 64108, USA

**Keywords:** Binding affinity, QSAR, BCRP, Polymorph, Mitoxantrone

## Abstract

**Background:**

A computation approach based on integrating high throughput binding affinity comparison and binding descriptor classifications was utilized to establish the correlation among substrate properties and their affinity to Breast Cancer Resistant Protein (BCRP). The uptake rates of Mitoxantrone in the presence of various substrates were evaluated as an in vitro screening index for comparison of their binding affinity to BCRP.

The effects of chemical properties of various chemotherapeutics, such as antiviral, antibiotic, calcium channel blockers, anticancer and antifungal agents, on their affinity to BCRP, were evaluated using HEK (human embryonic kidney) cells in which 3 polymorphs, namely 482R (wild type) and two mutants (482G and 482T) of BCRP, have been identified. The quantitative structure activity relationship (QSAR) model was developed using the sequential approaches of Austin Model 1 (AM1), CODESSA program, heuristic method (HM) and multiple linear regression (MLR) to establish the relationship between structural specificity of BCRP substrates and their uptake rates by BCRP polymorphs.

**Results:**

The BCRP mutations may induce conformational changes as manifested by the altered uptake rates of Mitoxantrone by BCRP in the presence of other competitive binding substrates that have a varying degree of affinities toward BCRP efflux. This study also revealed that the binding affinity of test substrates to each polymorph was affected by varying descriptors, such as constitutional, topological, geometrical, electrostatic, thermodynamic, and quantum chemical descriptors.

**Conclusion:**

Descriptors involved with the net surface charge and energy level of substrates seem to be the common integral factors for defining binding specificity of selected substrates to BCRP polymorph. The reproducible outcomes and validation process further supported the accuracy of the computational model in assessing the correlation among descriptors involved with substrate affinity to BCRP polymorph. A quantitative computation approach will provide important structural insight into optimal designing of new chemotherapeutic agents with improved pharmacological efficacies.

## Background

The computational tools intended for quantitative assessment of protein-ligand interactions are based on several factors including protein-ligand docking, molecular dynamic simulation and free energy calculations [1]. To better define the role of binding affinity in forming a protein-ligand complex, a structural characterization for putative human off-targets was recently performed on Nelfinavir, a potent HIV-protease inhibitor with pleiotropic effects in cancer cells [2]. In this experiment, they have adapted numerous computational models that integrated molecular dynamic simulation, free energy calculations with ligand binding site comparison and biological network analysis.

There are two integral screening approaches that could help identify and characterize the substrates and inhibitors of the efflux proteins and/or transporter system; the measurement of binding affinity and toxicity analysis of substrate compounds [3]. There was a report that drug resident time and uptake amount are better correlated with drug efficacy than the binding affinity [4-6], suggesting that lead optimization could be efficiently accomplished with analyzing the drug uptake profiles. Although numerous methodologies have been proposed for drug-target screening strategies based on binding affinity [7,8], there are no efficient computational tools available for the accurate estimation of the drug uptake profiles from the point of the molecular structures. In this study, the uptake rates of Mitoxantrone in the presence of various substrate compounds were examined as an in vitro screening index that could help to characterize the binding properties of chemotherapeutic drugs to tumor cells or efflux proteins.

Breast cancer resistant protein (BCRP) also known as ABCP or MXR or ABCG2 is a member of transporter super family ATP binding cassette (ABC) proteins. BCRP is known to affect the therapeutically available concentrations of various clinical agents [9-11]. Since the BCRP effluxes a wide range of structurally diverse xenobiotic compounds from cells [12], the broad distribution of BCRP not only renders less complete distribution of drugs but also causes a poor response of cells to chemotherapeutics [13-15]. BCRP in conjunction with P-gp expression at target sites affected the pharmacokinetic profiles of substrates and inhibitors [16]. Subsequently, the therapeutically available concentrations of certain agents increased in BCRP knock-out animal models that were highly prone to Mitoxantrone induced toxicity [17].

The in vitro studies on the BCRP efflux system have demonstrated that some cell lines displayed erratic efflux profiles of doxorubicin and rhodamine 123, and these observations were attributable to 482^nd^ position in the amino acid sequence consisting of arginine, glycine or threonine residues which are susceptible to numerous posttranslational modifications [18-20]. Three polymorphs, namely 482R (wild type) and two mutants (482G and 482T) of BCRP, have been identified, and alterations in their expressions and functions were reported [21]. Wild-type BCRP and its variants were markedly expressed in human embryonic kidney (HEK) cells [22].

The present study was intended to establish the relationships between chemical properties involved with the uptake rates of structurally diverse substrates and BCRP polymorphs. To achieve this goal, we have designed the computational model consisting of numerous molecular descriptors. The uptake rates of Mitoxantrone by BCRP were examined in the presence of various pharmacological classes of ABC transporter inhibitors, such as antiviral (i.e. Erythromycin, Foscarnet), antibiotic (i.e. Ciprofloxacin, Febendazole, Novobiocin, Quercitin), calcium channel blockers (i.e. Verapamil, Diltiazem, Nifedipine, Qunidine), anticancer (i.e. Mitroxantrone, Acyclovir, FTC, Phenethyl ITC, Raloxifene, Rodamin 123, Saquinavir, Tamoxifene), antifungal agents (i.e. Ketoconazole), hormones (i.e., Estradiol) and immunosuppressant (Cyclosporin) [16,23]. It was hypothesized that any changes in uptake rates of Mitoxantrone are due to competitive binding of these substrates to BCRP.

In the development of a computational model for prediction of structural specificity of substrate compounds to BCRP, three dimensional structures of the substrates were built using AMPAC with Graphical User Interface (Semichem, Shawnee Mission, KS). AMPAC used Austin Model 1 (AM1) for the quantum mechanical semi-empirical calculations of interactive energy. CODESSA can generate the numerical values for molecular descriptors, whereas the heuristic method (HM) preselects appropriate molecular descriptors. The multiple linear regression (MLR) is capable of deriving the linear QSAR based on them. The final outcomes were labeled as a characterization of compounds using derived properties X from AM1 calculations and regression with MLR, using measurements Y as response. The knowledge on such descriptors that determine substrate specificity to binding receptors is critical to delineate the drug interaction with BCRP polymorphs and the mechanisms behind their action. The outcomes of this study ultimately lead us to discover efficient new drugs with enhanced chemotherapeutic efficacies.

## Results and discussion

### The uptake rates of Mitoxantrone by HEK cells

**Figure 1:**
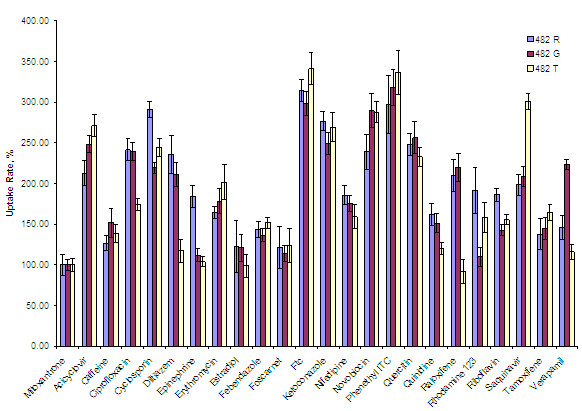
**Mitoxantrone uptake by the 482 R, 482 G and 482 T transfected HEK cells in the presence of various substrate compounds.** The data are expressed as mean +/− SD, p < 0.05, each experiment performed in quadruplicate.

The effects of various substrates on the uptake rates of Mitoxantrone by HEK cells were evaluated to determine their binding capacity to BCRP polymorphs. The uptake rates of Mitoxantrone (expressed per mg of protein) in the presence of various substrates were converted to the percentage uptake rate of Mitoxantrone in the absence of the substrates (Figure 1). The transcellular permeation profiles of the substrate compounds showed a similar trend to those of the uptake profiles, but statistical significance of the latter is much greater than the former. There are several important findings from this study.

1. Estrogen and tamoxifen did not significantly affect the Mitoxantrone uptake profiles, which are consistent with the previous findings [24].

2. Substrate compounds, such as Ciprofloxacin, Ketoconazole and Verapamil, allow for a greater Mitoxantrone uptake rate in 482G than 482R. BCRP substrates with the high binding affinities have common chemical structures, such as an azole ring [25], and quarternary nitrogen [26].

3. In HEK 482T, substrate compounds, such as Caffeine, Diltiazem, Epinephrine, Estradiol, Raloxifene and Verapamil, did not significantly affect the uptake rate of Mitoxantrone, whereas in both 482R and 482G, substrate compounds, such as Foscarnet and Rhodamine 123, did not significantly affect the uptake rate of Mitoxantrone.

It was suggested that changes in the uptake amount of Mitoxantrone in the presence of various substrates are due to their influence toward BCRP efflux polymorphs. Although an indirect approach associated with the uptake rate may not be the best option to predict the relationship with the chemical structures, the changes in the uptake rate of Mitoxantrone in the presence of substrate compounds could serve as a valid indicator for the drug affinity to BCRP.

### Relationships between the uptake rates and chemical properties

#### I. 482R Polymorphs

**Figure 2:**
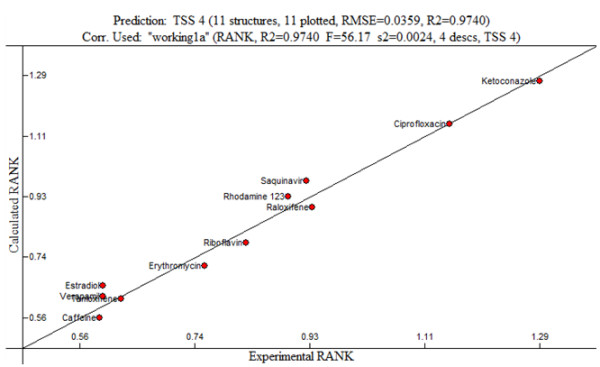
**a The best linear model for 482 R BCRP (R**^**2**^ **= 0.99, F = 460.38, cross validated R**^**2**^ **= 0.95). b**: The validation of the model on the testing data set.

The relationships between the uptake rates and the chemical structures of substrates were analyzed and quantitatively expressed as the concentration of a substrate required to exert a biological response. As shown in Table 1, four descriptors, HOMO-1 energy, Max atomic orbital electronic population, ESP-Max net atomic charge and average electrophilic reaction index for a O atom, mainly contribute to the linear relationship profiles in a QSAR model for 482R polymorph with the experimental coefficient value R^2^ of 0.9740 (F = 56.17 s^2^ = 0.024 Q^2^ = 0.8561: Table 2), which are indicative of a close correlation among them. As shown in Figure 2, the prediction power of the test substrates was also within 5% of the experimental value, which further corroborated the proposed model.

**Table 1:** The best linear models computed for 482 R, 482 G and 482 T of BCRP

**Type**	**The Best Linear Model**				
**482 R**	R^2^=0.9740	F=56.17	s^2^=0.024	Q^2^= 0.8561	(4, RANK)
		X	DX	t-test	
	0	-4.1145e+02	1.7878e+01	23.0141	Intercept
	1	1.2538e+01	6.6354e-01	18.8958	HOMO-1 energy
	2	2.9607e+02	9.1140e+00	32.4857	Max atomic orbital electronic population
	3	-1.0329e+01	5.3540e-01	19.2916	ESP-Max net atomic charge
	4	1.7837e+03	2.4776e+02	7.1996	Avg electroph. react. index for a O atom
**482 G**	R^2^=0.8455	F=82.1	s2=0.0181	Q^2^= 0.5986	(4, RANK)
		X	DX	t-test	
	0	-3.5513e+02	1.9119e+01	18.5750	Intercept
	1	-1.2124e+03	3.4838e+01	34.8023	ESP-Max net atomic charge 2
	2.	0790e+02	6.3673e+00	32.6508	Max SIGMA-PI bond order
	3	3.5144e+01	1.6146e+00	21.7656	Max 1-electron react. index for a C atom
	4	2.4821e+01	4.9829e+00	4.9812	ESP-FPSA-1 Fractional PPSA (PPSA-1/TMSA)
	[Quantum-Chemical PC]				
**482 T**	R^2^=0.8268	F=71.6	s2=0.027	Q^2^= 0.5617	(5, RANK)
		X	DX	t-test	
	0	2.0718e+02	4.8027e+01	4.3139	Intercept
	1	5.4190e+01	7.7933e+00	6.9535	ESP-Max net atomic charge
	2	6.1209e+01	2.6032e+00	23.5132	ESP-Max net atomic charge for a N atom
	3	8.9479e+00	6.5221e-01	13.7193	Number of double bonds
	4	2.0862e+00	2.3046e-01	9.0521	min(#HA, #HD) [Quantum-Chemical PC]
	5	7.9300e-01	1.9317e-01	4.1053	Min e-n attraction for a C-C bond

**Table 2:** **Computed R**^**2**^**and Q**^**2**^**values of the linear models for 482 R, 482 G and 482 T of BCRP**

	**Sets**	**R**^**2**^	**Q**^**2**^
482C	Training set	0.9740	0.8561
	Test set	0.9960	0.8506
482G	Training set	0.8455	0.5986
	Test set	0.9976	0.8336
482T	Training set	0.8268	0.5617
	Test set	0.9755	0.8438

It was noted that the dynamics of the aromatic cores and the alkyl tails can affect the electronic properties. The highest occupied molecular orbital (HOMO) and the lowest unoccupied molecular orbital (LUMO) are just two of molecular energy levels, and efficient energy conversion is an integral step in practical applications of substrates for charge transport and transfer processes through efflux proteins or transporters.

Another important descriptor seems to be overall net charge and surface charge of substrates. As the atomic charge of the molecule increases, the modulation of the uptake rate of Mitoxantrone decreases (X-axis is expressed as a negative absolute value), indicating that the charge in the molecules were inversely correlated with the uptake rates by BCRP 482R polymorphs. This finding is in a good agreement with the previous report that ABC transporters have a greater affinity to positively charged molecules which serve as an electron accepting functional group [27].

Phenethyl Isothiocyanate (PEITC), which showed a higher affinity to 482R, has an electropositive property originated from thiocyanate functional groups which may contribute to its strong affinity to 482R. On the other hand, Substrates, such as caffeine, estradiol and verapamil didn’t significantly affect the uptake rate of Mitoxantrone by 482R polymorphs.

#### II. 482G Polymorphs

**Figure 3:**
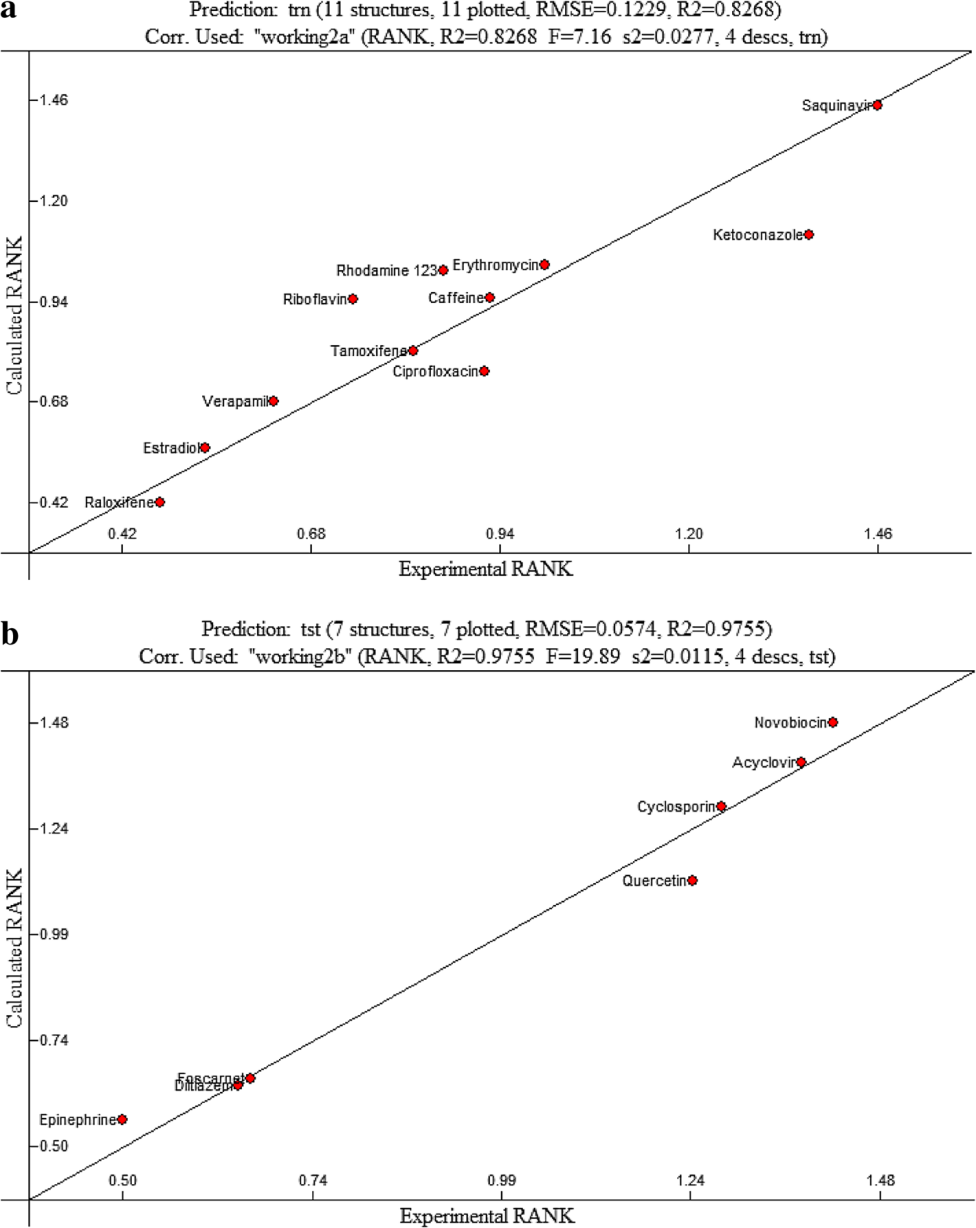
**a The best linear model for 482 T BCRP (R**^**2**^ **= 0.99, F = 238.48, cross validated R**^**2**^ **= 0.99). b**: The validation of the model on the testing data set.

The results of the study with 482G polymorph were most properly expressed in a linear QSAR equation consist of 4 descriptors, which are ESP-Max net atomic charge, Max SIGMA-PI bond order, Max 1-electron reaction index for a C atom, and ESP-FPSA-1 Fractional PPSA (PPSA-1/TMSA) [Quantum-Chemical PC], with the correlation coefficient for the modulated uptake of R^2^ of 0.8455 (F = 82.1 s2 = 0.0181 Q^2^ = 0.5986), as shown in Tables 1 and 2. The predicted value for the substrate molecules was also within 5% of the experimental value as shown in Figure 3. Similar to the results of 482R polymorph, it was also demonstrated that affinity of the 482G polymorph to BCRP decreases, as the charge on the molecule increases. The correlation coefficient between two variables (i.e., charge and binding affinity) for 482G polymorph is less significant than that of 482R.

Among substrates, Phenethyl Isothiocyanate (PEITC) has the highest binding affinity to 482G. The binding affinity of Rhodamine123 to 482G polymorph is lower than that to 482R. Febendazole and Riboflavin showed a low binding affinity to 482G, mainly due to the presence of multiple double bonds and aromatic rings. Substrates, such as Rhodamine123 (123%), Estradiol (114%) and Foscarnet (111%), did not significantly affect the Mitoxantrone uptake rate by 482R polymorph.

#### III. 482T Polymorph

As shown in Table 1, a linear QSAR for 482T polymorph consists of 5 descriptors; ESP-Max net atomic charge, ESP-Max net atomic charge for a N atom, Number of double bonds, min (#HA, #HD) [Quantum-Chemical PC], and Min e-n attraction for a C-C bond. The correlation value for the modulated uptake rate was 0.8268 (F = 71.6, s2 = 0.027, Q^2^ = 0.5617) and the predicted power of the test molecules was within 5% of the experimental outcomes, indicating that there is a good correlation among selected parameters. As shown in Figure 3, differing from the results of 482R and 482G polymorph, there is a positive relationship between surface charge and binding affinity; as a charge on the molecule increases, its binding affinity to 482 T polymorph increases. The results of this study suggest that the presence of the charged residue significantly affects the affinity of test substrates even though it does not substantially contribute to the specificity to each BCRP polymorph. The steric factors are likely to play a vital role in the relationship between substrate property and binding affinity, even though their contribution to the binding affinity of substrates to BCRP is much less than the charged residue.

482T polymorph has a lower affinity towards substrates, such as Caffeine, Diltiazem, Raloxifene, Quinidine and Verapamil. Fumitremorgin C (FTC) showed the highest impact on the uptake rate of Mitoxantrone by 482T, which is probably due to the presence of the charge species on FTC. Since the side effects of FTC (i.e., neurotoxicity) arises from stereo chemical constraints on the conformation of the diketopiperazine D ring, the replacement of the proline moiety (E ring) by an acyclic substituent might allow the adjacent diketopiperazine ring to assume a new conformation with the less charge species that renders the diastereoisomeric mixtures of FTC analogues less neurotoxic than native FTC [28,29].

Quercitin, which is known as the most active reactive oxygen species (i.e., peroxynitrite) scavenger among the structural analogues of flavonoids, had a significant impact on the uptake rate of Mitoxantrone in all three polymorphs, probably due to the fact that Quercitin has high resonance and donates electrons on the oxygen atom even though it lacks nitrogen atoms on the aromatic ring. It was reported that molecule oxygen atom on C-4 of C ring of Quercetin carries the largest excess charge, whereas charge accumulation on the hydroxy groups at the same ring is not considerably large [30,31]. On the other hand, estradiol did not affect the uptake rate of Mitoxantrone in all three polymorphs, indicating that estradiol is not a major substrate for BCRP.

The results of this study underline importance of complement regulatory proteins in the biologic systems that outline the binding capacity of exogenous compounds and subsequent their uptake rates.

#### IV. The model validation process

The Cross Validation process was carried out to confirm the predicting power of the QSAR model. The error values of the coefficient computed through QSAR model were obtained through assessment of percentage Absolute Relative Error (ARE) using the absolute value calculation of [(Actual Output - Predicted Output)/Actual Output].

**Figure 4:**
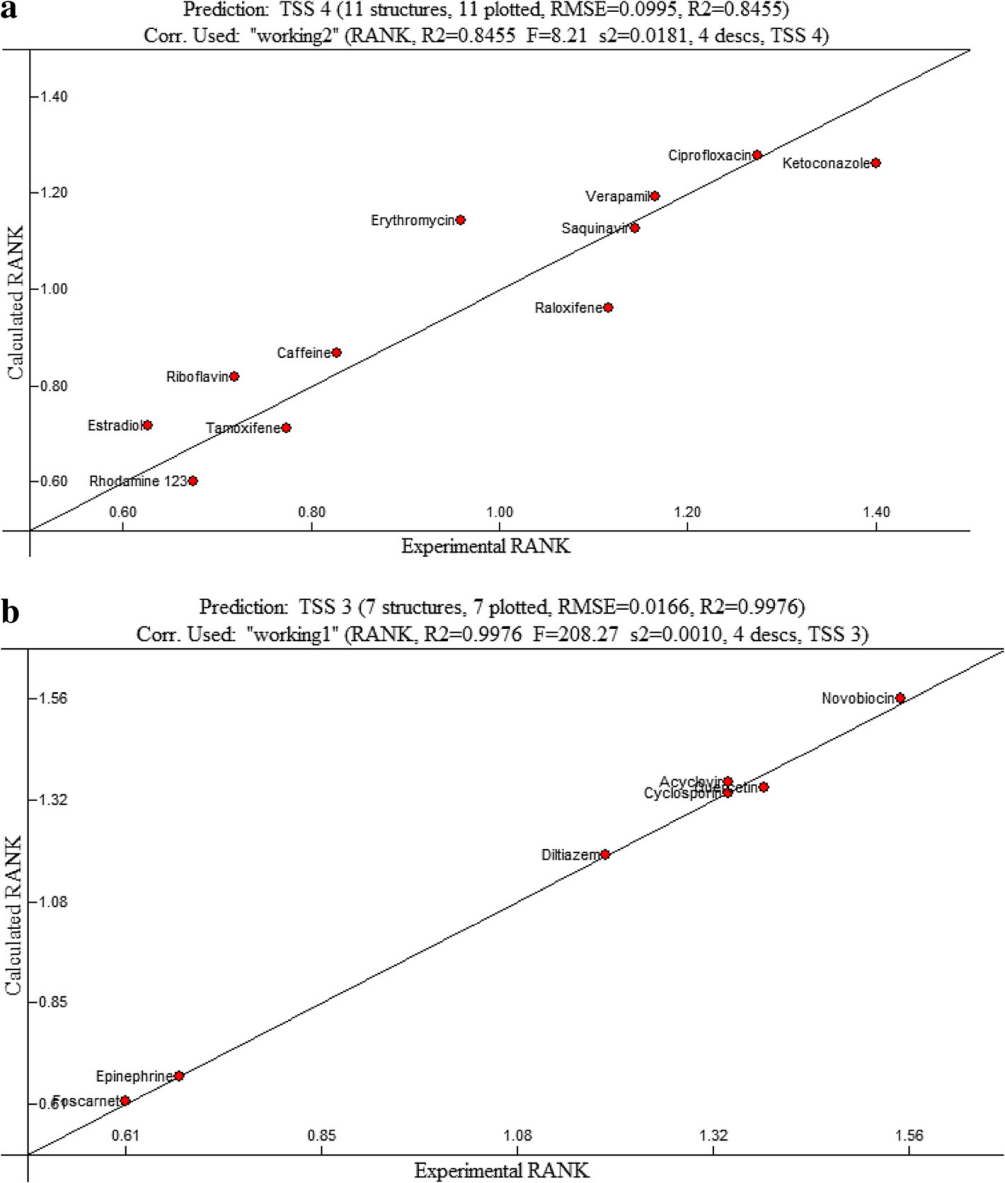
**a The best linear model for 482 G BCRP (R**^**2**^ **= 0.99, F = 576.86, cross validated R**^**2**^ **= 0.90).b**: The validation of the model on the testing data set.

The experimental and expected values of each compound were plotted for the validation process as shown in Figures 2b, 4b and 3b for 482R, 482G and 482T, respectively. The error values for both individual and combined descriptors were within 10% of the predicted values (Table 3), indicating that the predicted values from the linear model are in good agreement with the experimental values. It was also proved that the QSAR can accurately predict the effects of various substrates on the uptake rate of Mitoxantrone based on the given set of variables. The linear relationships with the experimental coefficient value (R^2^) and cross validate coefficient value (Q^2^) of 0.9 and 0.85 (Figure 2b), 0.99 and 0.83 (Figure 3b) and 0.97 and 0.84 (Figure 3b) for 482R, 482G and 482T, respectively, are indicative of a close correlation between experimental values and calculated values.

**Table 3:** Validation of the uptake rates of Mitoxantrone for the testing set of substrate compounds: Correlation between the experimental values of testing substrates and their calculated values based on the best linear model for each polymorph of BCRP

**TEST SET**	**482R**	**482R**	**482G**	**482G**	**482T**	**482T**
	**Exp. Uptake value**	**Cal. Uptake value**	**Exp. Uptake value**	**Cal. Uptake value**	**Exp. Uptake value**	**Cal. Uptake value**
**Acyclovir**	**212**	**160**	**248**	**230**	**274**	**312**
**Epinephrine**	**181**	**188**	**123**	**119**	**115**	**105**
**Foscarnet**	**126**	**111**	**111**	**135**	**128**	**115**
**FumitremorginC**	**305**	**361**	**291**	**406**	**327**	**391**
**Ketoconazole**	**270**	**272**	**258**	**266**	**263**	**204**
**PEITC**	**288**	**266**	**313**	**346**	**329**	**355**
**Quercetin**	**247**	**274**	**252**	**269**	**239**	**288**

The results of this study suggested that the binding affinity of substrates to specific receptors or efflux proteins is not entirely dependent on a particular variable or an individual group of variables in the relationship, but it is rather affected by various combinations of variables. This finding further supported the robustness of the QSAR approach in predicting the outcome from the medical database by avoiding a spurious association within a set of variables. It was also suggested that descriptors involved with the drug uptake profiles will give new insights on chemical modifications that can lead to designing new chemotherapeutic agent with improved pharmacological properties.

To design a pharmacologically active drug for site-specific activity is a challenging task that begins with rationally identifying the targets to which that drug binds. There are a number of computational approaches in designing efficient therapeutics based on target identification and lead optimization. As the specific binding to active targets may have a profound impact on the overall pharmacological activity [32], the effects of a variety pattern of protein binding reflected in the uptake profiles by cell membrane on therapeutic efficacy of drugs could be adapted as a primary screening means. The importance of protein binding has already been validated by less specific protein kinase inhibitors which attack tumors through multiple mechanisms [33]. This strategy has been effective to more than one type of cancer therapies.

A thorough understanding of drug binding interactions and their relationship with the biological activity requires high-throughput computational biology approaches. Computational techniques that identify competitive binding substrates and their inhibition range in cellular networks have been intensively developed, but their scales are very limited to initial assessment process [6,34]. Moreover, the qualitative description of the chemical entity currently available showed a limited predictive power due to the high dynamic nature of molecular structures and complicated responses from biological systems including complex efflux pathways. The mathematical modeling approaches, such as ordinary differential equations and pi-calculus, have limitations in that they require a large number of kinetics parameters to simulate the dynamic behavior of the biological system to which chemical entities bind [35,36]. Therefore, a functional dynamic model based on the qualitative descriptors defined from the competitive uptake profiles is integral for parameter optimization and dose regimen specification of new drug entities.

In recent years, major efforts have been placed on identifying and characterizing ABC transporters. They are expressed at the major barriers within the body (e.g., intestine, blood–brain barrier, placenta, kidney, and liver), where they lowered the uptake rate or enhanced the clearance of drugs [37]. Breast Cancer Resistant Protein (BCRP) is one of the most recently discovered members of ABC transporters. BCRP is a homo-dimer and consists of 655 amino acids containing a nuclear binding domain and a membrane spanning domain. BCRP shares broad similarities with bacterial, yeast, insect and other mammalian ABC transporter proteins [38]. In normal human tissues, BCRP was detected at higher levels in the placenta and at lower levels in the brain, prostate, ovary, colon, testis, liver, small intestine, kidney and heart [22,39]. Among normal tissues, BCRP is expressed in sycytiotrophoblasts of placenta, epithelium of small intestine, colon, liver, ducts and lobules of breast, and haemopoietic stem cells [22,40].

ABC transporters comprise various efflux proteins, some of which exert abnormal responses to exogenous compounds due to the presence of polymorphs. Nine polymorphs have been identified for MDR-1 [41]. In a clinical trial, ABCG2 polymorphism has a vital role in delineating the effective dose in chemotherapy [42]. Genetic polymorphism in ABC transporters influences numerous diseases including hypertension [43], lung cancer [44] and colon cancer [45]. The mutation of polymorphs discovered from human BCRP serves an integral criterion for the differential affinity of substrates to BCRP [46]. It was found that the affinity of BCRP to substrates can be modulated by altering the substrate specificity of multi-drug transporters. Several BCRP variants from direct DNA sequencing of the BCRP gene have been reported [47]. It was also demonstrated that single nucleotide polymorphisms (SNP) of BCRP produced individual variations in the pharmacokinetics and toxicity profiles of BCRP substrates. BCRP G34A (Val12Met) and C421A (Gln141Lys) polymorphisms occurred at high frequency in most ethnic populations and have been associated with the expression and activity of BCRP protein [48]. It has distinctive features including racial differences; for instance, BCRP V12M, Q141K, P269S and Q126Stop were detected in Korean at frequencies of 23, 28, 0.2 and 1.9%, respectively [49].

This study was undertaken to define various physicodynamic and chemical properties of substrates to BCRP polymorphs and elucidate the rationales behind their efficacies. The structurally diverse compounds were evaluated for elucidation of the BCRP polymorph mediated uptakes and establishment of the relationships with their molecular structures. The results of this study suggested that the chemical properties of exogenous compounds significantly influence BCRP polymorphs mediated uptake rate. All the compounds tested in this study are either substrates or inhibitors of at least one of BCRP polymorphs. The analysis on the chemical properties of the substrates based on the combining AMPAC/CPDESSA approach could help us to identify integral descriptors that should be mirrored by interactions with receptor proteins [50].

In this study, the consistent appearance of surface charge, electrophilic reactivity indices and molecular orbital energy descriptors obtained from the sets of chemotherapeutic substrate compounds supported the proven concept that charged electrophiles with the high energy level affected the affinity to BCRP polymorphs. The quantum chemical descriptors of the substrates, such as atomic orbital electronic population and bond order, also significantly contribute to its affinity to BCRP polymorph. It is known that the docking analysis of descriptors provides a qualitative representation of ligand and protein interactions in the QSAR model, even though the selection of docked conformations is often complicated due to its sensitivity to the scoring function. The results of this study demonstrate that substrate compounds containing net charged radicals can activate efflux proteins or peptides in the complement system. There is also a close correlation between descriptors and molecular weight, especially for bulky groups. The steric contour analysis indicates that the addition of bulky groups in the active region reduces the binding affinity.

It is possible that subjects with these polymorphisms may have different levels of single nucleotide polymorphisms (SNP) expression level and cellular localization and, consequently, varying degrees of efflux capability to model compounds [51]. Further studies are needed to determine which level and sites of SNP mainly contribute to the specificity of BCRP bindings. The findings in this study provide rationales behind the development of new drugs whose working mechanisms are closely correlated with substrate or inhibitor properties against BCRP polymorphs. The results of this study can lead to detailed constitutional descriptors that can be directly translated to a chemical structure, such as connectivity indices and descriptors describing substitution patterns. It is possible to combine and translate calculated properties of descriptors into a new chemical/pharmaceutical entity through the visualization process by the contour map and an analyzing tool like GaussView program (GaussView 3.07: Gaussian Inc., Wallingford, CT). It is certain that numerous training compounds need to span through the model fitting techniques, addressing not only finding a fit, but also the predictive feature of the fit. While the outcomes of this study have not directly steered us to a new compound, they have helped us to identify important structural insight into optimal designing of new chemotherapeutic agents. Recently, a drug class called poly ADP ribose polymerase (PARP) inhibitors that targets cancers caused by BRCA mutations have shown promise in clinical trials treating breast cancer [52].

## Conclusions

In summary, the chemotherapeutic effects of the known substrates were classified based on their binding affinity to BCRP. The computational approach with the sequential approaches of Austin Model 1 (AM1), CODESSA program, heuristic method (HM) and multiple linear regression (MLR) was performed to derive QSAR model and its predictive power was validated. The BCRP mutations may induce conformational changes as manifested by the altered uptake rates of Mitoxantrone by BCRP in the presence of other competitive binding substrates that have a varying degree of affinities toward BCRP efflux. At the practical level, the use of a computational structural approach will help the scientists identify the best compound and its linear dose range with improved pharmacological efficacy, eliminating the need to perform multiple assays over a wide range of concentrations in defining the binding affinity and uptake rates.

## Methods

HEK (human embryonic kidney) cell lines transfected with each polymorph (i.e., 482R, 482G and 482T) were kindly donated by NIC (NIH, Bethesda, MD) [53]. The minimum essential medium was purchased from ATCC (Manassas, VA). Penicillin, Streptomycin, and Geneticin were purchased from Invitrogen (Carlsbad, CA). Radioactive Mitoxantrone was obtained from American Radiolabelled Inc (St Louis, MO). All other chemicals and testing compounds were obtained from Sigma (St Louis, MO).

### Cell culture preparation

HEK cell lines transfected with 482R, 482G and 482T plasmids were grown in the minimum essential medium supplemented with 10% FBS, 50 IU/ml penicillin, 50 μg/ml streptomycin, 4 mM L-glutamine and 100 nM Geneticin. Cells were incubated in 75 mm^2^ plastic culture flasks at 37°C supplemented with 5% CO_2_/95% air. BCRP expression in HEK cells was confirmed by RT-PCR using the method previously described [54].

### A drug uptake study

HEK cells were trypsinized and loaded in 24 well plates at a seeding density of 2 × 10^5^ cells per well. Cell viability was maintained by providing a fresh medium every other day until they reached confluence. Cells were exposed with radioactive Mitoxantrone (~100 μM) in the presence or absence of various inhibitors at specific concentrations or 10 μM Fumitremorgin C **(**FTC) (as a positive control) at 37°C for 5 min. The drug uptake process was stopped by washing the cells with 1 ml of ice-cold DPBS for 3 times, followed by lysis with the Triton X/0.1 M NaOH solution. A cell digest (100 μl) was taken and diluted to 5 ml with 30% Scintisafe™ (Fisher Scientific, NJ). The cumulative amount of Mitoxantrone in diluted samples was determined using Beckman Coulter Counter and expressed as the percentage amount of the control. Mitoxantrone accumulation was normalized for cellular protein and presented as the percentage of the control, where the control represents cells treated with Mitoxantrone in the absence of any inhibitors. Data were expressed as mean +/− SD, p < 0.05 and by one way ANOVA.

### A drug permeability study

HEK cell lines were prepared as described previously [54]. After confluence was achieved, the TEER value of cells was measured to verify the presence of tight junction. The growth medium was replaced and washed with PBS. The radioactive Mitoxantrone (~100 μM) in the presence or absence of inhibitors was added on the basolateral side of the transwells. The samples were collected from the apical side at predetermined time intervals for up to 120 min. Apparent drug permeability (Papp) value was calculated using the formula Papp = (dQ/dt)/(A x D_0_), where (dQ/dt) is the linear appearance rate of drug in the apical side, A is the cross-sectional area of the Transwell insert and D_0_ is the initial concentration of the compound in the baso-lateral compartment [55]. The experiment was repeated for 4 times for each inhibitor (N = 4).

### The selection of datasets

**Figure 5:**
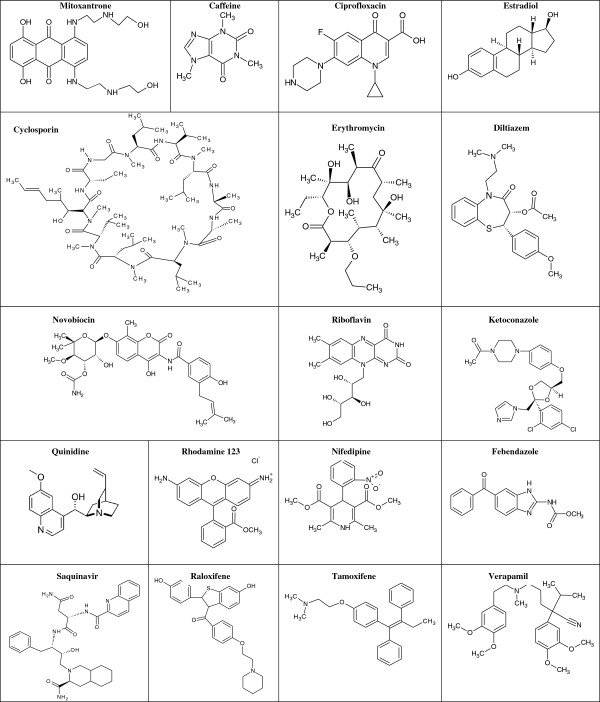
Structures of Mitoxantrone and substrates tested in this study.

As shown in Table 4, the substrates selected according to the different pharmacological categories were evaluated for their effects on the flux rates of Mitoxantrone. The experimental sets (25) were divided into 18 training sets and 7 test sets (i.e., for validation purpose). The structures of the substrates in the training set are shown in Figure 5. The uptake rates for Mitoxantrone or those in the presence of the substrates obtained from the experimental transport study were considered to be directly proportional to the affinity and/or permeation rate of drugs with BCRP.

**Table 4:** The changes in the uptake rate of Mitoxantrone in 482 R, 482 G and 482 T BCRP transfected HEK cell line in the presence of substrate compounds

		**Changes in Uptake of Mitoxantrone (cm/sec: %)**		
		**482R**	**482G**	**482T**
	**Mitoxantrone**	23.71 (100%)	27.43 (100%)	25.92 (100%)
**Training Set**	**Caffeine**	29.64 (125%)	41.42 (151%)	46.28 (140%)
	**Ciprofloxacin**	57.38 (242%)	63.91 (233%)	45.88 (177%)
	**Cyclosporin**	70.18 (296%)	66.93 (244%)	63.76 (246%)
	**Diltiazem**	55.95 (236%)	59.52 (217%)	32.40 (125%)
	**Erythromycin**	37.93 (160%)	48.00 (175%)	50.02 (193%)
	**Estradiol**	29.87 (126%)	31.27 (114%)	26.70 (103%)
	**Febendazole**	30.82 (130%)	34.83 (127%)	38.36 (148%)
	**Ketoconazole**	64.50 (272%)	70.22 (256%)	68.17 (263%)
	**Nifedipine**	38.17 (161%)	42.52 (155%)	38.36 (148%)
	**Novobiocin**	56.90 (240%)	77.35 (282%)	71.02 (274%)
	**Quinidine**	38.64 (163%)	41.97 (153%)	33.17 (128%)
	**Raloxifene**	46.47 (196%)	55.95 (204%)	23.58 (91%)
	**Rhodamine 123**	44.57 (188%)	33.74 (123%)	43.02 (166%)
	**Riboflavin**	41.25 (174%)	35.92 (131%)	36.80 (142%)
	**Saquinavir**	46.00 (194%)	57.33 (209%)	72.83 (281%)
	**Tamoxifene**	31.30 (132%)	38.67 (141%)	40.95 (158%)
	**Verapamil**	29.87 (126%)	58.42 (213%)	31.36 (121%)
**Test Set**	**Acyclovir**	50.26 (212%)	66.91 (244%)	68.95 (266%)
	**Epinephrine**	42.91 (181%)	33.73 (123%)	24.89 (115%)
	**Foscarnet**	29.87 (126%)	30.45 (111%)	33.17 (128%)
	**FumitremorginC**	26.76 (305%)	79.82 (291%)	84.75 (327%)
	**Ketoconazole**	64.02 (270%)	70.77 (258%)	68.17 (263%)
	**PEITC**	68.28 (288%)	85.85 (313%)	85.27 (329%)
	**Quercetin**	58.56 (247%)	69.12 (252%)	61.95 (239%)

The data are expressed as mean +/− SD, p < 0.05, each experiment performed in quadruplicate.

### A QSAR approach

This approach was intended to select the most suitable descriptors among the descriptor sets for defining binding specificity of each polymorph. Three dimensional structures of the substrates were built using AMPAC with Graphical User Interface (Semichem, Shawnee Mission, KS) [56]. For structural classification of descriptors, AMPAC used Austin Model 1 (AM1), which is the Hamiltonian widely utilized in the quantum mechanical semi-empirical calculations of interactive energy. AM1 achieved energy minimization of the gradient norm (0.05 kcal/mol) using 20 simplex iterations followed by 1000 steps of Powell minimization [57]. CODESSA (Comprehensive Descriptors for Structural and Statistical Analysis; Semichem, Shawnee Mission, KS) is an advanced, full featured Quantitative Structure/Activity Relationship (QSAR) program that connects information from AMPAC to experimental data [58]. CODESSA preselects each subset of structure descriptors, which include constitutional, topographical, geometrical, electrostatic, thermodynamic and quantum chemical properties, as shown in Table 5. CODESSA can generate the numerical values for up to 600 molecular descriptors which can be used for the regression analysis [59].

**Table 5:** Descriptors used in this study for identifying the best linear model

**Class**	**Name of Descriptors**
**Constitutional**	Number of double bonds
	Number of aromatic bonds
	Number of Oxygen atoms
	Average distance sum connectivity index
	ESP-RNCS: Relative negative charged SA (SAMNEG*RNCG)
	ESP-RPCS Relative positive charge SA (QMPOS/QTPLUS)
	[Zefirov’s PC]
	WPSA3 weighted PPSA (PPSA3*TMSA/1000)
	[Zefirov's PC]
	ESP-FPSA-1 Fractional PPSA (PPSA-1/TMSA)
**Thermodynamic**	Translational Entropy
	Total Entropy
	Total Enthalpy
**Electrostatic**	Max partial charge
	Max partial charge for a hydrogen atom
	Max partial charge for a carbon atom
**Quantum Chemical**	Max atomic orbital electronic population
	ESP-Max net atomic charge
	Avg electroph. react. index for a O atom
	Max 1-electron react. index for a C atom
	Max SIGMA-PI bond order
	ESP-Max net atomic charge
	ESP-Max net atomic charge for a N atom
	min(#HA, #HD) [Quantum-Chemical PC]
	Min e-n attraction for a C-C bond
	Min e-n attraction for a C-H bond
	HOMO-1 energy (Molecular orbital related)
	LUMO + 1 energy (Molecular orbital related)

The heuristic method (HM) preselected appropriate molecular descriptors and derived the linear QSAR model based on them. Those descriptors that exist for all molecules in the training set were included, whereas those descriptors whose values did not vary throughout the training set were excluded from the regression. The number of descriptors in the final QSAR models was usually less than one third of the number of molecules in the data set [60]. HM allows us to obtain the best QSAR based on F and t test values, which were set in such way that descriptors having more than 0.99 and less than 0.8 correlations were excluded as they may generate an over-optimistic regression.

Molecular descriptors selected by the heuristic method (HM) in CODESSA were used as inputs to perform multiple linear regression (MLR), which is the simplest method that builds a single regression equation for a given data set. For each regression analysis, the goodness of fit was evaluated by examining the number of molecules (*N*), coefficient of determination (r^2^), cross-validated standard error (Q^2^) and value of the *F*-statistic (*F*). The Q^2^ value obtained through the leave-one-out algorithm (LOO) cross-validation procedure reflects the stability of the model through perturbation of the regression coefficients with the acceptability criterion of 0.5 in most CoMFA studies [61].

### The model validation process

The linear QSAR model was cross validated using the error values acquired in the prediction process [62]. To optimize the validation outcomes, a relationship between the experimentally obtained values and computed uptake rates from the test set was established using the heuristic method. The expected values for each compound were calculated and plotted to elucidate their correlations with the experimental values.

Each descriptor was assigned a number through the web based random number generator ( http://www.random.org) for cross validation. Individual descriptors were sequentially incorporated into the regression process to monitor the error value. The following two-fold cross-validation scheme was implemented.

1. The experimental data (25) were divided into 2 subsets; 18 training sets and 7 test sets for the model validation.

2. The data from the selected subsets were categorized into the descriptors listed in Table 5.

3. The odd ratio for each descriptor was calculated to find the most potential contributors to binding property with defined risk weights.

4. The significance of the final regression is determined by comparing prediction Absolute Relative Errors (AREs) which are obtained using the absolute value of [(Actual Output - Predicted Output)/Actual Output] to the test subset. An estimated p value less than 0.05 was considered to be significant for the consistency of the model. The model with the lowest prediction error generated through the cross validation process was chosen to represent the best outcome for each BCRP polymorph.

## Competing interests

The authors declare that they have no competing interests.

## Authors’ contribution

YL designed the study and drafted the manuscript. SJ performed the computational modelling process and statistical analysis. GA carried out the cell culture, drug binding and immunoassay. CHL conceived of the study, participated in its coordination and helped to complete the manuscript. All authors read and approved the final manuscript.
